# Natural Killer T Cells Activated by a Lipopeptidophosphoglycan from *Entamoeba histolytica* Are Critically Important To Control Amebic Liver Abscess

**DOI:** 10.1371/journal.ppat.1000434

**Published:** 2009-05-15

**Authors:** Hannelore Lotter, Nestor González-Roldán, Buko Lindner, Florian Winau, Armando Isibasi, Martha Moreno-Lafont, Artur J. Ulmer, Otto Holst, Egbert Tannich, Thomas Jacobs

**Affiliations:** 1 Bernhard-Nocht-Institute for Tropical Medicine, Hamburg, Germany; 2 Division of Structural Biochemistry, Research Center Borstel, Leibniz-Center for Medicine and Biosciences, Borstel, Germany; 3 Unidad de Investigación Médica en Inmunoquímica, Hospital de Especialidades del Centro Médico Nacional Siglo XXI del Instituto Mexicano del Seguro Social (IMSS), Mexico City, Mexico; 4 Departamento de Inmunología, Escuela Nacional de Ciencias Biológicas, Instituto Politécnico Nacional, Mexico City, Mexico; 5 Division of Immunochemistry, Research Center Borstel, Leibniz-Center for Medicine and Biosciences, Borstel, Germany; 6 Immune Disease Institute and Department of Pathology, Harvard Medical School, Boston, Massachusetts, United States of America; 7 Division of Immunology and Cell Biology, Research Center Borstel, Leibniz-Center for Medicine and Biosciences, Borstel, Germany; University of Wisconsin-Madison, United States of America

## Abstract

The innate immune response is supposed to play an essential role in the control of amebic liver abscess (ALA), a severe form of invasive amoebiasis due to infection with the protozoan parasite *Entamoeba histolytica*. In a mouse model for the disease, we previously demonstrated that Jα18^-/-^ mice, lacking invariant natural killer T (*i*NKT) cells, suffer from more severe abscess development. Here we show that the specific activation of *i*NKT cells using α-galactosylceramide (α-GalCer) induces a significant reduction in the sizes of ALA lesions, whereas CD1d^−/−^ mice develop more severe abscesses. We identified a lipopeptidophosphoglycan from *E. histolytica* membranes (EhLPPG) as a possible natural NKT cell ligand and show that the purified phosphoinositol (PI) moiety of this molecule induces protective IFN-γ but not IL-4 production in NKT cells. The main component of EhLPPG responsible for NKT cell activation is a diacylated PI, (1-*O*-[(28∶0)-*lyso*-glycero-3-phosphatidyl-]2-*O*-(C16:0)-Ins). IFN-γ production by NKT cells requires the presence of CD1d and simultaneously TLR receptor signalling through MyD88 and secretion of IL-12. Similar to α-GalCer application, EhLPPG treatment significantly reduces the severity of ALA in ameba-infected mice. Our results suggest that EhLPPG is an amebic molecule that is important for the limitation of ALA development and may explain why the majority of *E. histolytica*-infected individuals do not develop amebic liver abscess.

## Author Summary

Amoebiasis is a widespread human parasitic disease caused by the intestinal protozoan *Entamoeba histolytica*. There are two major clinical manifestations of the disease, amebic colitis and amebic liver abscess. Interestingly, only a small proportion of *E. histolytica*-infected individuals develop invasive disease, whereas the majority harbors the parasite within the gut without clinical symptoms. So far, cells of the innate immune system have been described to constitute the main host defense mechanism for the control of amoebiasis, relying largely on the early production of interferon-γ (IFN-γ), a protein, which activates macrophages to kill microorganisms including parasites. However, the IFN-γ-producing cells as well as the amebic antigen involved in the activation have not been identified. Here we demonstrate that mice challenged with live *E. histolytica,* and which are deficient of a specific lymphocyte population known as natural killer T cells (NKT cells), have a reduced capacity to control ameba infection and develop much larger amebic liver abscesses compared to normal mice. In addition, we isolated a molecule from the surface membrane of *E. histolytica*, which constitutes a lipopeptidophosphoglycan, and which activates NKT cells for the production of protective IFN-γ. Thus, our study provides a mechanism for the innate control of ameba invasion that might explain why the majority of *E. histolytica*-infected individuals do not develop amebic disease.

## Introduction

*Entamoeba histolytica* the causative agent of human amebiasis is an intestinal protozoan parasite that causes significant morbidity and mortality worldwide [1]. The main symptoms associated with ameba infection arise when the parasite breach the colonic mucosa, leading to severe hemorrhagic colitis or the development of extraintestinal abscesses, most commonly in the liver. Interestingly, only a small proportion of individuals infected with *E. histolytica* develop invasive amebiasis while the majority harbors the parasite in the gut without clinical signs of disease [2],[3]. From *in vitro* studies as well as from animal models for experimental amebic liver abscess (ALA) it is well documented that IFN-γ plays an important role in the early control of *E. histolytica* invasion. The development of amebicidal activity by neutrophils and monocytes *in vitro* is dependent on IFN-γ [4]–[7]. Accordingly, the depletion of IFN-γ by monoclonal antibodies or the targeted disruption of the IFN-γ receptor in mice led to more severe tissue destructions of the liver parenchyma in the mouse model for ALA [8],[9]. Effector lymphocytes with innate like functions such as γδ–T cells, natural killer cells or natural killer T (NKT) cells infiltrate into the center of experimental ALA and thus can serve as a source for the protective IFN-γ in the early phase of abscess development. Hence, mice deficient for γδ-T cells, but more importantly, Jα18^−/−^ mice, lacking NKT-cells, have been shown to develop considerably larger abscesses compared to respective controls [8]. NKT cells are involved in immune responses in a broad range of diseases, including autoimmunity, allergy, cancer and infectious diseases [10]. Murine NKT cell populations are heterogeneous [11], but the majority expresses an invariant Vα14−Jα18 ΤCR and therefore they are referred to as invariant NKT (*i*NKT) cells, that unlike activation of conventional αβ−T cells by antigenic peptides, recognize glycolipids presented by the nonclassical antigen presenting molecule CD1d. Upon ligation of their TCR, *i*NKT cells can produce large amounts of a variety of cytokines with sometimes opposite function, including the pro-inflammatory IFN-γ as well as the anti-inflammatory IL-4, IL-10 and IL-13, which is believed to instruct the development of subsequent immune responses. The prototypical and by far most studied *i*NKT cell antigen is the glycolipid α-galactosylceramide (α-GalCer), a marine sponge glycolipid, which is a potent CD1d-restricted agonist widely used for *in vitro* and *in vivo* experiments to decipher *i*NKT cell function [12]. In recent years, it has been shown that *i*NKT cells can be activated directly by recognition of microbial glycolipids presented by CD1d or, indirectly by soluble mediators such as IL-12 and/or by recognition of endogenous ligands, both provided by dendritic cells (DC) s−stimulated *via* Toll-like receptors (TLRs) [10],[13]. In addition to α-GalCer, diverse natural CD1d ligands that stimulate *i*NKT cells have been identified in various microorgansims [14]–[18]. Accordingly, mice lacking NKT cells and in particular *i*NKT cells, have an increased susceptibility to various bacterial, fungal, and parasitic infections [8],[15],[17],[19].

Similar to other protozoa, *E. histolytica* exposes on its surface a complex GPI-anchored glycoconjugate, designated *E. histolytica* lipopeptidophosphoglycan (EhLPPG) [20]. As virulent and non-virulent amebae differ in the amount and the antigenicity of their LPPG these molecules have been associated with pathogenicity [21]–[24]. The structural analysis of EhLPPG revealed the presence of the Gal_1_Man_2_GlcN-*myo*-inositol motif linked to a phosphoserine backbone substituted by the linear carbohydrate chains [Glcα1-6]_n_Glcβ1-6Gal. Although a lipid anchor was proposed for this molecule, evidence concerning the structure of the lipid has not been provided so far. Thus, information is lacking whether this structure may facilitate a CD1d restricted activation of NKT cells in a CD1d restricted manner. However, toll-like receptor pathways were involved in the induction of IL-12, IL-8, IL-10 and TNFα by EhLPPG [25].

The study presented here was aimed to further investigate the role of NKT cells in the development of ALA, to isolate and characterize the structure of a potential natural ligand from *E. histolytica* trophozoites and to analyse the mechanism of NKT cell activation by dendritic cells presenting EhLPPG.

## Results

### Control of amebic liver abscess requires presentation of glycolipids by CD1d

**Figure 1:**
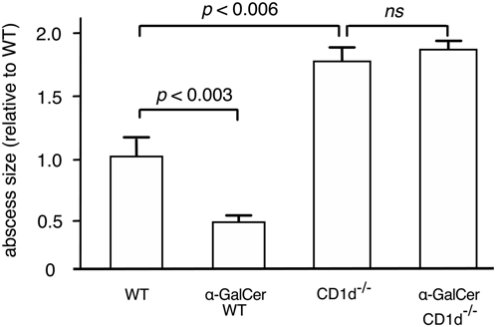
Role of NKT cells in the control of ALA in mice. Mice treated with α-GalCer or mice lacking CD1d (CD1d^−/−^) were intrahepatically infected with virulent *E. histolytica* trophozoites. Seven days p.i. animals were sacrificed and sizes of liver abscesses determined. Bars represent sizes of abscess scores relative to those of wildtype control mice (WT). Results were obtained from 3 independent experiments comprising 5 to 7 animals each (statistics: Mann-Whitney U test).

In a previous study we have shown that mice lacking V_α_14-J_α_18 *i*NKT cells have a reduced capacity to control experimentally induced amebic liver abscess (ALA). The lack of control in these knock out mice was evidenced by substantially larger abscess sizes and increased re-isolation rates of *E. histolytica* trophozoites from the liver lesions when compared to wild type controls [8]. To further investigate the role of *i*NKT cells in the control of ALA, we specifically activated *i*NKT cells by using the non-physiological ligand α-GalCer. Wild-type C57BL/6 mice were treated with a single dose of α-GalCer, 24 h prior to ameba challenge. This treatment resulted in a significant reduction of ALA lesions (p<0.003). In contrast, ALA in CD1d^−/−^ mice, lacking *i*NKT and *d*NKT cells, were significantly increased, irrespectively whether or not treated with α-GalCer (p<0.006) (Fig. 1). The results indicate the importance of NKT cells in the control of abcess formation.

### Isolation of LPPG from *E. histolytica* trophozoites

**Figure 2:**
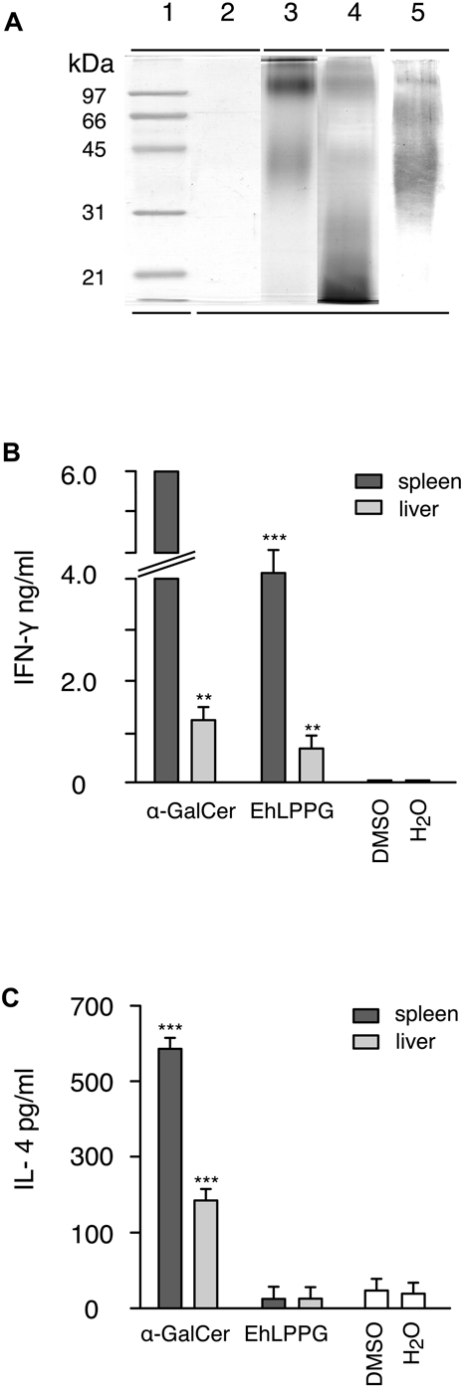
EhLLPG purified from membranes of *E. histolytia* trophozoites stimulates production of IFN-γ but not of IL-4 by splenocytes and liver lymphocytes. (A) EhLPPG was separated by a 12% SDS-PAGE and developed by the following staining procedures: colloidal Coomassie (lane 2), periodic acid-Schiff staining (lane 3), a modified silver nitrate staining (lane 4) [61] and a Western-blot of EhLPPG developed with mAb EH5 (lane 5) [28]. A low molecular weight protein standard (BioRad), stained with colloidal Coomassie, is shown in lane 1. The negative stain in lane 2 revealed the abscence of major protein content, a positive staining in lane 3 and 4 evidenced the high glycosylation of the molecule and the detection by EH5 in lane 5 proved the identity of *E. histolytica* LPPG. (B,C) APC (1x10^5^) were pulsed for 3 h with 2 µg α-GalCer or EhLPPG prior to co-cultivation with spleen or gradient separated liver lymphocytes (4x10^5^). Supernatants were taken after 48 h and tested for the presence of IFN-γ (B) and IL-4 by ELISA (C). IFN-γ produced compared to DMSO control ^*^( *p*<0.05); ^**^( *p*<0.005), ^***^( *p*<0.001); Mann Whitney U-test. The results were obtained from experiments repeated at least 5 times.

The finding that CD1d is required to limit ALA development suggested that an ameba glycolipid that is presented by CD1d and which is able to activate *i*NKT cells is involved. A likely candidate is the *E. histolytica* LPPG (EhLPPG), which is present in considerable quantities on the surface of *E. histolytica* trophozoites. Accordingly, EhLPPG was isolated using an adaptation of the original reported method [26]. The EhLPPG recovered from the aqueous phase after hot phenol-water extraction was initially characterized by 12% SDS-PAGE gels and was visualized by different staining procedures (Fig. 2A). The purified EhLPPG yielded a negative staining with colloidal Coomassie-blue (lane 2), which detects as low as 30 ng of protein content. Positive reaction with the periodic acid Schiff (PAS) reagent (lane 3) or silver nitrate (lane 4) evidenced a high degree of glycosylation of the molecule resulting in a broad band with two major molecular mass regions between 97–200 kDa and 30–65 kDa, which was in agreement with previous reports [23],[27]. The presence of EhLPPG was further confirmed by a Western-blot developed with EH5 (lane 5), a monoclonal antibody specific for EhLPPG [28].

### Induction of IFN-γ by EhLPPG

In order to examine whether the purified EhLPPG was able to stimulate lymphocytes from wild-type C57BL/6 mice *in vitro*, antigen-presenting cells (APC) were generated and pulsed with α-GalCer or purified EhLPPG prior to co-cultivation with isolated liver or spleen lymphocytes. Supernatants were analysed for the presence of IFN-γ and IL-4, respectively. Stimulation of spleen or liver lymphocytes with α-GalCer or EhLPPG resulted in significant IFN-γ production (Fig. 2B). However, similar to recently described microbial glycolipids [17], the stimulation with EhLPPG reached only 30–50% of the IFN-γ levels induced by α-GalCer, possibly due to the exceptional strong affinity of the non-physiological ligand α-GalCer [29]. In contrast to α-GalCer, EhLPPG-induced lymphocytes did not produce significant amounts of IL-4 (Fig. 2C). The LPS content in the amebic EhLPPG preparations was below 0.25 EU/ml at an EhLPPG concentration of 2 µg/ml that induced significant levels of IFN-γ. This LPS concentration did not induce cytokine production in control experiments (data not shown).

### Isolation and structural characterization of the phosphatidylinositol moiety from EhLPPG

**Figure 3:**
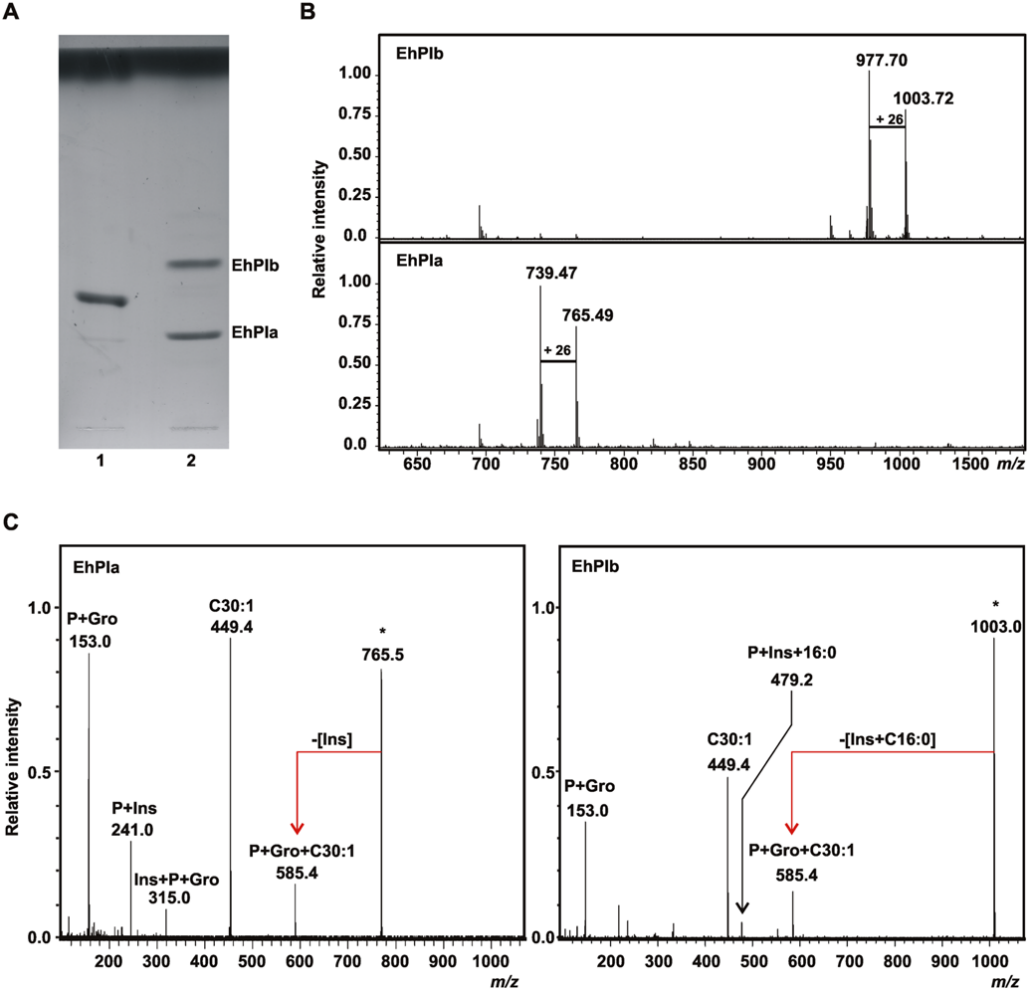
Structural characterization of the PI moieties of EhLPPG. (A) Thin-layer chromatography. Lane 1, PI from Sigma, lane 2 shows the two isoforms EhPIa and EhPIb obtained after nitrous acid deamination ofEhPI. (B) EhPIs were analyzed by ESIFT-ICRMS. Negative ion mass spectrum of EhPIa (upper) and EhPIb (lower). The [M]-26 corresponded to the exchange in the use of a 30∶1 or a 28∶1 fatty acid. EhPIs were further analyzed by ESI-IRMPD. (C) Negative ion mass spectrum of the major parent ions found in EhPIa and EhPIb shown in (B).

**Figure 4:**
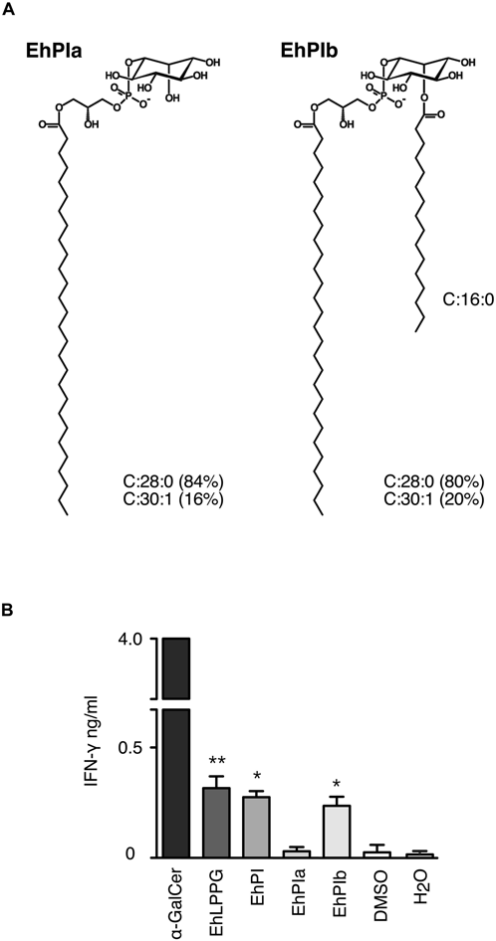
Structure and NKT cell stimulatory capacity of EhPIs. (A) The proposed structures of EhPIa (left) and EhPIb (right). The only difference between the EhPIs is the acylation of inositol at position 2 in EhPIb. The fatty acids and the molar ratios are indicated. (B) IFNγ production of NKT cells stimulated by APCs pulsed with α-GalCer, EhLPPG, EhPI, EhPIa and EhPIb. IFN-γ production compared to DMSO control ^*^( *p*<0.05);^**^( *p*<0.005); ANOVA, Dunnett. The results were obtained from three independent experiments.

To further characterize the portion of EhLPPG that might be responsible for lymphocyte activation, and in particular to determine whether the phosphatidylinositol moiety of the GPI anchor from EhLPPG (EhPI) is involved, EhPI was separated from EhLPPG after cleavage by nitrous acid deamination. HPTLC analyses demonstrated the presence of two products, designated EhPIa and EhPIb, respectively (Fig. 3A). These bands were isolated and analyzed by GC-MS after methanolysis and peracetylation in order to determine the chemical composition. Both EhPIa and EhPIb contained glycerol (Gro), Inositol (Ins), as well as the fatty acids 30∶1, 28∶0 and 16∶0, respectively, the latter being present only in minor amount in EhPIa. ESIFT-ICRMS mass spectrometry revealed the presence of two prominent pseudomolecular ions at *m/z* 765.49 and *m/z* 739.47 in EhPIa, and at *m/z* 1003.72 and *m/z* 977.70 in EhPIb, evidencing a difference of [M]+26 *m/z* in both cases. The most abundant ions in EhPIa and EhPIb at *m/z* 739.47 and *m/z* 977.70, respectively, corresponded to the molecules containing 28∶0, whereas the ions at *m/z* 765.49 (EhPIa) and *m/z* 1003.72 (EhPIb) corresponded to the molecules containing 30∶1 (Fig. 3B). The fine structural characteristics of both molecules were deduced after ESI-IRMPD experiments (Fig. 3C). For EhPIa, the presence of the daughter ion at *m/z* 241.0 (inositol-1,2-cyclic phosphate) identified the parent ion as a PI and further indicated that the inositol ring was not acylated at the position 2 [30]. The additional presence of only one fatty acid carboxylate ion at *m/z* 449.4 (30∶1) and the abundant presence of the ion at *m/z* 153.0 (cyclic glycerophosphate) identified this PI as a *lyso*-acyl anchor [31]. The ions at *m/z* 585.4 and *m/z* 315.0 corresponded to P+Gro+30∶1 and Ins+P+Gro, respectively. In the case of EhPIb, the virtual absence of the ion at *m/z* 241.0 and the presence of the pseudomolecular ions at *m/z* 585.4 (showing the loss of the Ins+16∶0 fragment) as well as at *m/z* 479.2 (P+Ins+16∶0), demonstrated a substitution with 16∶0 at position 2 of the Ins. Acylation of inositol was the only structural feature that differentiated EhPIb from EhPIa. Similar to EhPIa, the abundant presence of the ion at *m/z* 153.0 in EhPIb and the strong fatty acid carboxylate ion at *m/z* 449.4 (30∶1), indicated a *lyso*-acyl anchor in this PI isoform as well [31]. Based on these results, the *E. histolytica* LPPG lipid anchors were assigned as 1-*O*-(28∶0)-*lyso*-glycero-3-phosphatidylinositol for EhPIa and 1-*O*-[(28∶0)-*lyso*-glycero-3-phosphatidyl-]2-*O*-(16∶0)-inositol for EhPIb (Fig. 4A). Interestingly, stimulation of lymphocytes in the presence of EhLPPG or separated EhPIa and EhPIb, respectively, indicated that EhPIb, but not EhPIa, is the active portion of EhLPPG that contains the capacity to induce the production of IFN-γ (Fig. 4Β).

### EhLPPG activates NKT cells in a CD1d-restricted manner

**Figure 5:**
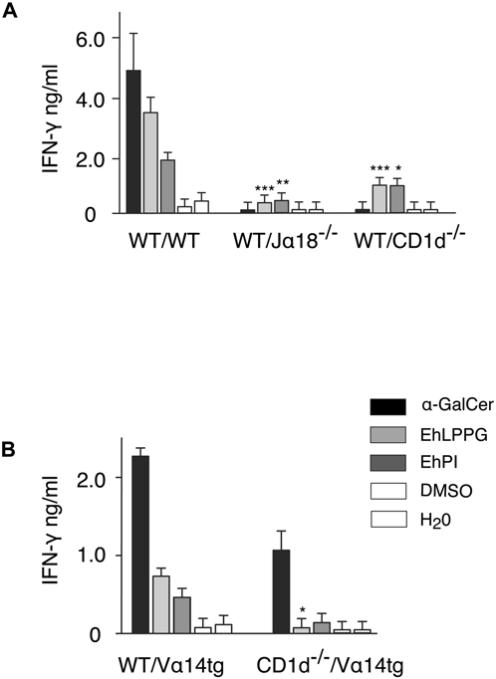
CD1d-restricted NKT cell activation by EhLPPG and EhPI. (A) APC (5x10^4^) from WT mice were pulsed with either 2 µg of α-GalCer, EhLPPG or EhPI and incubated with 1x10^5^ lymphocytes of spleen cell preparations from Jα 18^−/−^ lacking *i*NKT cells and CD1d^−/−^ mice lacking *i*NKT and *d*NKT cells. IFN-γ secretion was measured by ELISA. Difference of IFN-γ production in EhLPPG/EhPI activated WT splenocytes ^*^( *p*<0.05);^**^( *p*<0.005),^***^( *p*<0.001); ANOVA, Dunnett. (B) APC were generated from either WT or CD1d^−/−^ mice, pulsed as described above and incubated with gradient purified liver lymphocytes from Vα14-transgenic mice. Liver *i*NKT cells were further purified by magnetic cells sorting. IFN-γ was assessed by ELISA. Difference of IFN-γ production in EhLPPG/EhPI activated WT splenocytes ^*^( *p*<0.05); student t test. The results were obtained from three independent experiments.

To determine whether indeed NKT cells are responsible for IFN-γ secretion after stimulation with EhLPPG or EhPI, lymphocytes were prepared from Jα18^−/−^ or CD1d^−/−^ mice lacking *i*NKT or all NKT subpopulations, respectively, and co-cultivated with EhLPPG- or EhPI-pulsed APC from wild-type C57BL/6 mice. Cells from both Jα18^−/−^ and CD1d^−/−^ mice revealed a strong reduction in IFN-γ secretion, indicating that *i*NKT cells represent the major source for IFN-γ. To further investigate CD1d-restriction of EhLPPG-mediated NKT-cell activation, APC from wild type or CD1d^−/−^ mice were pulsed with α−GalCer, EhLPPG and EhPI, respectively, and co-cultivated with purified T cells from liver or spleen of V_α_14 tg mice (Fig. 5B). As expected, in contrast to APC from wild type mice, APC from CD1d^−/−^ mice were impaired in their ability to activate NKT cells. Purified T cells incubated with α-GalCer, EhLPPG and EhPI does not induce IFN-γ in the abscence of APC (data not shown). Taken together, these results suggest that EhLPPG and in particular EhPI activates *i*NKT cells to produce IFN-γ in a CD1d restricted manner.

### EhLPPG-induced NKT cell activation requires toll-like–receptor 2/6 and functional IL-12

**Figure 6:**
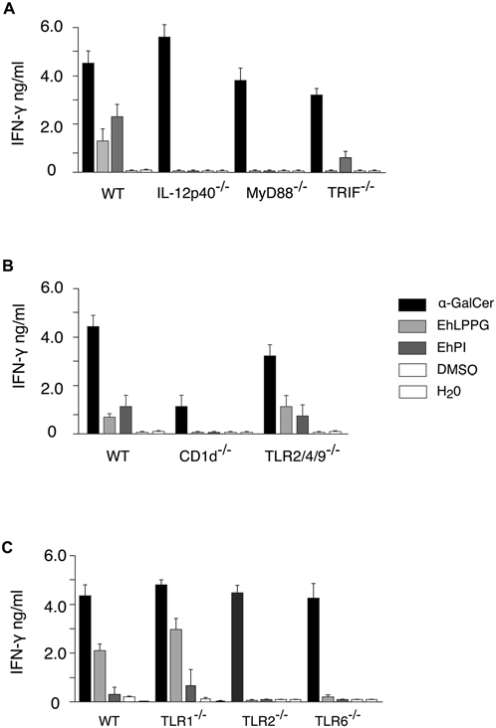
Activation of NKT cells by EhLPPG involves molecules of the TLR signalling pathway. APC from IL12-p40^−/−^, MyD88^−/−^, TRIF^−/−^ (A), CD1d^−/−^, TLR2^−/−^, 4^−/−^, 9^−/−^ (B), and TLR1^−/−^, 2^−/−^ and 6^−/−^ (C) were generated, pulsed with EhLPPG and used to stimulate purified T cells from Vα14tg mice. The production of IFN-γ by purifed T cells was determined by ELISA. EhLPPG-dependent NKT cell activation was abrogated in CD1d^−/−^, IL-12p40^−/−^, MyD88 and TLR 2^−/−^ and TLR 6^−/−^ mice. The results were obtained from two independent experiments.

To determine whether CD1d-restricted *i*NKT cell activation by EhLPPG requires the involvement of Toll-like-receptor (TLR) pathways, APC from knock out mice lacking the TLR-related adapter molecules MyD88 and TRIF, respectively, were pulsed with EhLPPG or EhPI and co-cultivated with T cells from V_α_14 tg mice (V_α_14*i*NKT). In addition, parallel experiments were performed with APC from mice lacking TLR1, TLR2 or TLR6, or with APC that are impaired to produce functional IL-12 (IL-12p40^−/−^) (Fig. 6). The results indicated that a TLR pathway other than TLR3 is required for *i*NKT cell activation, as APC generated from MyD88^−/−^ but not from TRIF*^−/−^* mice were unable to induce IFN-γ by V_α_14*i*NKT cells. Moreover, the finding that IFN-γ is induced by APC from TLR1^−/−^, but not by those generated from TLR2^−/−^ and TLR6^−/−^ mice suggested that EhLPPG or EhPI were capable to activate APC by binding to TLR2, TLR6 or to TLR2-TLR6 heterodimers. In addition to TLR2 and/or TLR6 signaling, *i*NKT cell activation by EhLPPG or EhPI required the presence of functional IL-12, as APC from IL-12p40^−/−^ mice were incapable to induce IFN-γ production by V_α_14*i*NKT cells.

We did not detect IL-4 in the supernatants of the co-culture experiments using APC from the various knock-out mutants pulsed with EhLPPG and iNKT cells (data not shown).

### EhLPPG is internalized by APC

**Figure 7:**
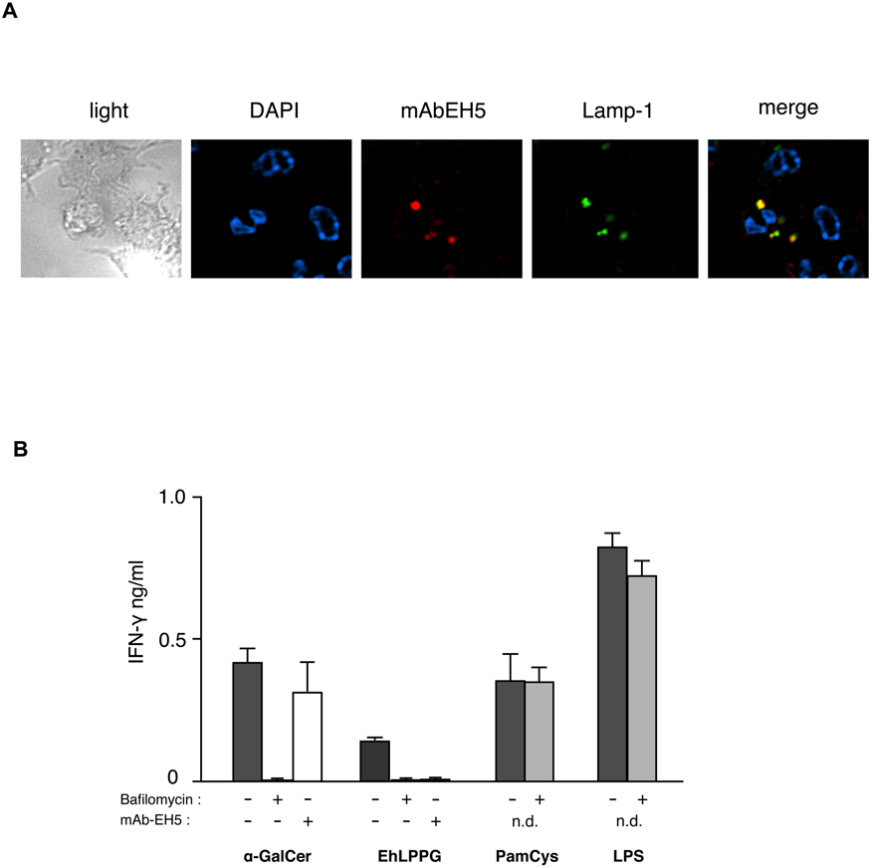
EhLPPG is internalized and requires processing or endosomal trafficking for activation of NKT cells. (A) WT APC were pulsed with 20 µg/ml EhLPPG for 3 h, fixed with 4% PFA and permeabilized with 0.5% saponin; EhLPPG was labeled with mAb EH5 [28] and anti Lamp-1 mAb was used for detection of late endosomes by confocal microscopy. The experiment was performed four times. (B) Treatment of APC with an inhibitor of endocytosis, bafilomycin A1 (10 nM, 30 min), led to a decrease in the IFN-γ production in NKT cells after stimulation with α-GalCer (4 µg/ml) and EhLPPG (10 µg), but not with PamCys (4 µg/ml) and LPS (1 ng/ml). The addition of mAb EH5 (100 µg/ml) to APC pulsed with α-GalCer and EhLPPG specifically inhibited the IFN-γ secretion of cocultured NKT cells.

To gain more insight into the CD1d-restricted activation process we performed several experiments to evaluate if EhLPPG directly binds to CD1d without further processing. To this end EhLPPG was incubated with plate bound CD1d and purified NKT cells. However, no activation of NKT cells was observed. Also CD1d-tetramer incubated with EhLPPG was unable to stain NKT cells (data not shown). These data suggest that EhLPPG cannot bind directly to CD1d molecules. In order to analyse whether EhLPPG enter the endocytic pathway which would allow further processing as a prerequisite for presentation on CD1d, we performed immunofluorescence analysis using the monoclonal anti-EhLPPG antibody EH5. Here we found that the ameba lipopeptidophosphoglycan is internalized as anti-EH5 staining was found to co-localize with Lamp-1 positive vesicles (Fig. 7A). Thus, EhLPPG was targeted to lysosomes and/or late endosomes which would allow processing and loading of the molecule to CD1d. To corroborate this finding we inhibited the internalization and processing of antigens by pre-incubation of APC with bafilomycin A1, which interferes with the uptake of macromolecules in endosomes [32]. The inhibition of endocytosis abrogated the IFN-γ production of NKT cells by EhLPPG and α-GalCer, but not the IFN-γ production induced by the TLR-specific activators PamCys or LPS used as positive controls [33],[34]. In addition the incubation of antigen-pulsed APC with the mAb EH5, but not with an isotype control (data not shown), inhibited the IFN-γ production of NKT cells in response to EhLPPG (Fig. 7B). This may indicate that the mAb EH5 recognizes EhLPPG presented on CD1d and thus prevented binding to the T cell receptor. Collectively these data indicate that a processing of the EhLPPG molecule is necessary to allow its presentation via CD1d.

### EhLPPG treatment reduces the severity of ALA

**Figure 8:**
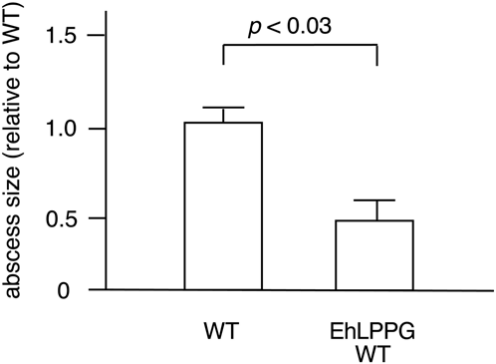
Development of ALA in EhLPPG treated mice. 24 h prior to intrahepatic challenge with virulent *E. histolytica* trophozoites, WT mice were treated with 4 µg EhLPPG by i.p. application. Seven days post infection, mice were sacrificed and sizes of abscesses were determined. Shown are sizes of ALA in EhLPPG treated mice relative to untreated controls. Results were obtained from 2 independent experiments comprising 5 to 6 animals each (statistics: Mann-Whitney U test).

To investigate whether EhLPPG similar to α-GalCer is able to inhibit ALA development wildtype mice were treated with purified EhLPPG 24 h prior to intrahepatic challenge with virulent *E. histolytica* trophozoites. The results clearly indicate significant protection in EhLPPG treated mice compared to respective controls (Fig. 8).

## Discussion

The majority of individuals infected with *E. histolytica* harbor the parasite within the gut without any clinical signs of disease. Only a small proportion may develop invasive amebaisis, e.g. amebic colitis or amebic liver abscess (ALA). In this study we report on the isolation and characterization of an *E. histolytica* lipopeptidophosphoglycan (EhLPPG) that activates *i*NKT cells which were previously shown to be crucial in the control of ALA in a mouse model for disease [8]. EhLPPG was found to be a potent stimulator of NKT cells to produce IFN-γ, but not IL-4. Structural characterization revealed that a particular phosphatidylinositol (EhPI) isoform present within the GPI anchor of EhLPPG constitutes the active component for IFN-γ induction. Dissection of the pathway involved in NKT cell activation indicated that direct recognition of EhLPPG or EhPI presented on CD1d was not sufficient for NKT cell activation, as it also required TLR signaling and IL-12 production.

The importance of NKT cells for the control of ALA *in vivo* was previously shown by the use of Jα 18^−/−^ mice lacking *i*NKT cells [8] and further confirmed in this study by the finding that CD1d^−/−^ mice lacking both *i*NKT and *d*NKT cells are more susceptible to ALA development. In addition, application of the most potent and specific NKT cell activator, α-GalCer reduced sizes of ALA significantly. Likewise, mouse models for malaria, trypanosomiasis or cryptococcus infection have shown that a single treatment with α-GalCer rapidly stimulated IFN-γ production by *i*NKT cells and reduced parasite and bacterial burden, respectively [19],[35],[36].

EhLPPG, which is expressed on the surface of *E. histolytica* trophozoites [20],[23],[27],[37] was identified as the ameba molecule that activates *i*NKT cells in vitro and which is able to reduce ALA development when applied to mice prior to amebic challenge. To this end we isolated the EhLPPG by an improvement of the original method [26]. This method reduced the possibility of contamination with phospholipids and small glycolipids that could be co-extracted with LPPG and thus may interfere with/or compete for the binding to CD1d, or may potentially provide an indirect stimulus by ligation of TLR. A basic biochemical characterization demonstrated the highly glycosylated nature of the purified compound, which was further identified as EhLPPG by its reactivity with the EhLPPG-specific monoclonal antibody EH5 [28]. EhLPPG is structurally related to glycoconjugates dominating the cell surfaces of other protozoa like *Trypanosoma brucei*, *T. cruzi*, *Leishmania* and *Plasmodium.* Previous studies on EhLPPG in which the structure was partially characterized indicated the presence of the consensus sequence Gal_1_Man_2_GlcN-*myo*-inositol that corresponds to the core region of GPI anchored molecules, although the nature and arrangement of the lipid portion linked to the inositol moiety remained unclear [27]. In the study presented, it is demonstrated that the EhLPPG is in fact a GPI anchored molecule, applying biochemical degradation, gas chromatography and mass spectrometry of its PI moieties. From these results it is concluded that *E. histolytica* trophozoites posses two isoforms of PI, EhPIa and EhPIb, respectively (Fig. 3A). In EhPIa, the glycerol is only substituted at the *sn*-1 position by a single, long fatty acid chain (28∶0 or 30∶1), similarly to LPG from *Leishmania major* [38]. EhPIb bears the same substitution with 28∶0 or 30∶1 at the *sn*-1 of glycerol but interestingly had an additional 16∶0 in the inositol ring, which is not a common feature among protozoa [39], although it was found in *Plasmodium falciparum* GPI [40] and in *T. brucei* PARP GPI, where the glycerol is monoacylated as well as the inositol is substituted by 16∶0 [41]. This non-common fatty acid distribution might confer particular biological properties to EhLPPG in the interaction with components of the innate immune system like CD1d molecules. These molecules belong to a family of major histocompatibility antigen-like molecules that bind gycophosphinositol with a high affinity and regulate the function and differentiation of NKT cells [42].

*In vitro* stimulation of APC by EhLPPG induced secretion of IFN-γ by NKT cells. However, compared to α-GalCer, stimulation with EhLPPG was significantly weaker, similar to levels obtained with glycolipid preparations from other microorganisms such as bacteria or protozoa [16]–[18]. The strong induction of IFN-γ by α-GalCer might be due to the composition and length of the alkyl (C26:0) and sphingosine (C18:0) chains, which appear to be optimal for CD1d binding [43]. On the other hand and in contrast to α-GalCer, EhLPPG did not induce secretion of IL-4. Although the mechanism for the induction of IL-4 is not well understood, a recent investigation suggested that the length of the lipid chain plays an essential role since the truncation of the sphingosine chain promoted the induction of IL-4 and triggered a Th2 immune response [44].

Activation of *i*NKT cells due to microbial infections can be achieved through direct or indirect pathways (for rev. see [10]). The direct, cognate or antigen mediated activation requires the uptake of the glycolipid, processing and subsequent loading to CD1d molecules in the endosomes of the APC. In this scenario, *i*NKT cells are engaged via their invariant TCR recognizing antigen presented on CD1d without any contribution of additional cytokines released from the APC. Beside α-GalCer as the classical direct activator of *i*NKT cells, glycolipids from various bacteria or protozoa have the capacity to activate NKT cells by CD1d restriction independent from cytokines provided by the APC [15]–[18]. For indirect or adjuvant-like activation of *i*NKT cells, different possible pathways have been proposed. One of the indirect pathways involves the recognition of PAMPS by TLR resulting in the induction of IL-12 and/or IL-18 activating NKT cells in a CD1d-independent manner [45]. During *Salmonella typhimurium* infection, the NKT cell response depends on IL-12 and additionally on CD1d recognition [46]. Another activation mechanism has been described for LPS which induces IFN-γ production in NKT cells via IL-12 and IL- [34]. A third alternative is an indirect activation pathway induced by parasite eggs. Here endogenous glycolipid is upregulated by a yet unknown mechanism and presented via CD1d to NKT cells. So far, this mechanism has been described only for DCs sensitized with eggs from *Schistosoma mansoni* [47]. From the results presented here using a series of knock-out mice we conclude that activation of *i*NKT cells by EhLPPG or EhPI requires both presentation of EhPI by CD1d as well as TLR signalling and IL-12 production.

In addition, we provide evidence that endocytosis and processing of EhLPPG is a prerequisite for CD1d-dependent NKT cell activation since i) we could not find a direct binding of EhLPPG to recombinant CD1d, ii) EhLPPG was found to co-localize with Lamp-1 as a marker for late endosomes which would allow processing and loading of the molecule to CD1d and iii) we showed that NKT cell activation is abrogated when APC endocytosis was inhibited by bafilomycin [48]. In addition, the incubation of APC with the mAb EH5 inhibited the IFN-γ production of NKT cells in response to EhLPPG. The finding that induction of IFN-γ by APC from TLR2^−/−^ and TLR6^−/−^ mice is abrogated suggests that EhLPPG and EhPI is capable to activate the APC by binding through these TLR. This is in agreement with recent results that diacylated motifs, as also present in the active EhPIb isoform, bind to TLR2-TLR6 heterodimers, while triacylated molecules engage TLR2-TLR1 heterodimers [49],[50]. However, we can not exclude that the inability of EhLPPG and recombinant CD1d to stimulate NKT cells is due to a lack of costimulatory molecules and/or cytokines. Moreover, the lack of direct binding of EhLPPG to CD1d might indicate that the observed activation of NKT cells by EhLPPG also involves an increased presentation of self-antigens by CD1d.

Interestingly, we did not find activity with the monoacylated EhPIa isoform, although such a structure is in principle capable to activate NKT cells via TLR2 and CD1d dependent pathways as shown for a structurally related LPG from *Leishmania* [15],[51]. In consideration of an adjuvant effect of TLR-ligands during activation of CD1d-restricted NKT cell, traces of contaminating lipopeptides could also be responsible for the TLR2-dependent response [52]. However, the double extraction method (chloroform methanol water and phenol water) used for the purification of EhLPPG should minimize the risk of contamination [53].

Our data demonstrate that early IFN-γ production by NKT cells is responsible for a sufficient control of parasites in the liver. In addition, NKT cells provide a link between innate and adaptive immunity due to their capacity to early produce large amounts of IFN-γ and IL-4 that can bias the immune response into either a TH1-or TH2 direction. The exclusive IFN-γ production of EhLPPG activated NKT cells can be expected to trigger the subsequent adaptive immune response into a TH1 type that would provide additional IFN-γ. This might augment efficient abcess control by T cell dependent mechanisms at later time points.

Taken together the results presented here indicate that EhLPPG is able to limit ALA development most likely due to its ability to specifically activate *i*NKT cells to produce IFN-γ. The importance of IFN-γ for the control of *E. histolytica* has been documented in various investigations [4],[5],[7],[8],[9],[54]. In particular, the mouse model used in this study recently revealed that the application of IFN-γ - neutralizing antibodies abrogates ALA development. Thus EhLPPG constitutes an ameba molecule that is critically important to control ALA and which might be responsible for the lack of amebic disease in the majority of *E. histolytica* infected individuals.

## Materials and Methods

### Cultivation of *E. histolytica*

Trophozoites of the *E. histolytica* isolate HM-1:IMSS were grown axenically in TYI-S-3 medium [55]. To maintain virulence, trophozoites were regularly passaged through the liver of C57BL/6 mice as described previously [8].

### Origin of mice

Wild-type C57BL/6 (WT), Vα14-Jα18 transgenic (tg), TLR1^−/−^, TLR6^−/−^, CD1d^−/−^ and Jα18^−/−^ were bred and housed under specific pathogen-free conditions at the Bernhard Nocht Institute for Tropical Medicine (Hamburg). IL12p40^−/−^; TLR2^−/−^ and MyD88^−/−^ were kindly provided by Christoph Hölscher, Research Center Borstel, Germany. TRIF^−/−^ were kindly provided by Bruce Beutler, La Jolla, California. All mice were backcrossed on a B6 genetic background for >10 generations.

### Experimental amebic liver abscess and treatment with α-GalCer

Amebic liver abscesses were induced by direct intrahepatic inoculation of virulent *E. histolytica* trophozoites as previously described [56] with minor modifications for the use of C57BL/6 mice [8]. The influence of α-GalCer and EhLPPG on the abscess formation was investigated by intraperitoneal application of 2 µg α-GalCer (Alexis, Axxora) or 4 µg EhLPPG diluted in PBS/0.05% Tween 20 per animal 24 h prior to amebic challenge. In our previous work we found that abscesses are self-limited and were resolved until day 21 post infection. Performing a kinetic analysis of abcess formation we found that day seven post infection is the most appropriate time point for studying the influence of immune mechanisms on abcess size [8]. Therefore, on day seven post intrahepatic inoculation of *E. histolytica* trophozoites, the animals were sacrificed and the size of the abscess lesions were measured in mm, a score was introduced and related to the score of ALA found in WT mice (score: 0 =  no abscess; 1 = <1 mm; 2 = 1–5 mm; 3 = >5 mm).

### Isolation of EhLPPG and its phosphatidylinositol (EhPI) moiety

Trophozoites of the late logarithmic phase of growth were washed, resuspended in pyrogen free water and lysed by freeze and thawing. The homogenate was centrifuged at 430 *g* at 4°C for 10 min and subsequently the supernatant was recovered and ultracentrifuged at 150,000 *g* for 40 min [57]. The obtained pellet was extracted with a mixture of chloroform/methanol/water 10∶10∶3 (by volume) and the insoluble material was recovered by centrifugation, dried, resuspended in distilled pyrogen free water and extracted three times with an equal volume of 90% phenol at 68°C for 30 min with constant stirring [58]. The water phase containing EhLPPG was recovered after centrifugation at 12,000 *g* for 30 min and dialysis against distilled water. In order to obtain the EhPI moiety, nitrous acid deamination was performed as described [59]. In brief, dried EhLPPG was resuspended in a mixture of 0.3 M sodium acetate buffer at pH 4.0 and 1 M sodium nitrite, incubated at 37°C for 2 h. The released EhPI moiety was recovered from the organic phase after partition between water and water-saturated 1-butanol. The organic phase was then dried under a stream of nitrogen and resuspended in a mixture of chloroform/methanol/water 10∶10∶3 (by volume) for analysis on Silica Gel 60 high-performance thin-layer chromatography (HPTLC) plates. EhPIa and EhPIb were separated on a preparative HPTLC and re-extracted with a mixture of Chloroform/methanol/water 10∶10∶3 (by volume). For the use in cell stimulation, PI, PIa and PIb were dried under a stream of nitrogen and resuspened in PBS/Tween (0.05%). EhLPPG and EhPI samples contained <0.25 endotoxin units (EU) per ml at the concentrations used for cell activation, as determined by the *Limulus* amoebocyte lysate assays (Cambrex).

### Immunochemical and spectroscopic analysis

EhLPPG (10 µg/lane) was analyzed by 12% SDS-PAGE and either stained to evidence the presence of protein (Coomassie brilliant blue) and carbohydrates (silver nitrate and periodic acid Schiff), or transferred to a PVDF membrane for Western blot analysis and subsequently developed with the LPPG-specific monoclonal antibody EH5 [28]. For compositional analyses, EhLPPG and EhPI were subjected to methanolysis. 150 µl of 0.5 M hydrochloric acid in dry methanol was added and the solution incubated at 85°C for 1 h, followed by addition of 50 µl of both pyridine and acetic anhydride and analyzed by GC-MS on a Hewlett Packard GL 5890 Gas chromatograph equipped with an Ultra-1 column (Agilent) and coupled to an electron impact mass detector.

Electrospray Ionization Fourier Transform Ion Cyclotron Mass Spectrometry (ESI FT-ICR MS) was performed in the negative ion mode using an APEX Qe – Instrument (Bruker Daltonics, Billerica, USA) equipped with a 7 Tesla actively shielded magnet. Mass spectra were acquired using standard experimental sequences as provided by the manufacturer. Samples were dissolved at a concentration of ∼10 ng/µl in a 50∶50∶0.001 (v/v/v) mixture of 2-propanol, water, and triethylamine and sprayed at a flow rate of 2 µl/min. Capillary entrance voltage was set to 3.8 kV, and dry gas temperature to 150°C.

Infrared-multiphoton dissociation (IRMPD) of isolated parent ions was performed with a 35 W, 106 µm CO_2_ laser (Synrad, Mukilteo, WA). The unfocused laser beam was directed through the center of the ICR cell and fragment ions were detected after a delay of 0.5 ms. The duration of laser irradiation was adapted for each sample to generate optimal fragmentation and varied between 10–80 ms.

### Generation of APC

Bone marrow was harvested from femurs of 6- to 10-week-old mice and cultured as described by Lutz et al. [60]. Cultures were supplemented with supernatants from Ag8653 myeloma cells transfected with the gene coding for murine GM-CSF (kindly provided by B. Stockinger, NMRI, Mill Hill, London, UK). The percentage of mature cells was determined by FACS analysis using anti- CD11c - APC, anti - CD40-FITC, anti - CD86-PE or anti - CD80 (BD-Bioscience) and ranged from 20–23%.

### Isolation of cells from spleen and liver

Spleens were perfused with hypotonic ammonium chlorid solution for erythrolysis. Subsequently, cells were washed twice with medium and adjusted to the appropriate number. Livers were perfused with ice cold PBS/20% FCS solution and subsequently filtered through a 40 µm mesh. Following centrifugation at 400 *g*, cell pellets were resuspended in RPMI-20 medium, underlayed with a 30% Nycodenz solution (Nycoprep^TM^, Universal) and centrifuged at 900 *g* for 20 min. The liver lymphocytes were collected from the interface, treated with hypotonic ammonium chloride solution, washed and subjected to magnetic bead sorting using the Pan T-cell isolation kit (Macs, Myltenyi). NKT-cell populations were analyzed for purity by flow cytometry using α-GalCer (Alexis; Axora) loaded CD1d-tetramer-PE (Proimmune) and anti-CD4-FITC (BD Bioscience). Purified NKT cells did not produce IFN-γ after cultivation with α-GalCer in the abscence of aditional APC.

### Co-culture of APC with NKT cells isolated from spleen or liver

BMDC were used as antigen presenting cells (APC). In brief, 4×10^4^ cells/well were cultured in triplicates in 96-well round bottom plates with RPMI-20 medium, supplemented with FCS, L-glutamine, antibiotics, sodium pyruvate. The cells were pulsed with α-GalCer or purified amebic EhLPPG or EhPI at indicated concentrations for 3 h. NKT-cell enriched spleen and liver lymphocytes were then added to the pulsed APC with 1×10^5^ cells/well and incubated for 24 h (IL-4) and 48 h (IFN-γ). Cytokines were measured with the respective sandwhich ELISA for IL-4 and IFN-γ (R&D System). To block the processing and presentation pathway, APCs were treated with 10 nM bafilomycin A1 (Sigma) 30 min before incubation with EhLPPG, α-GalCer, PamCys-SKK (EMC, Germany) or LPS (*E. coli*, serotype 055∶B5; SIGMA). In an additional set of experiments APC were pulsed for 3 h with EhLPPG or α-GalCer. Subsequently APC were incubated with 100 µg/ml mAb EH5 [28] before the addition of NKT cells.

### Intracellular localization of EhLPPG

APC from WT BL6 mice were pulsed with 20 µg/ml of EhLPPG and seeded in 24-well plates with glass coverslips and incubated with 5%CO_2_ at 37°C for 3 h. Subsequently, cells were washed, fixed with 4% paraformaldehyde and permeabilized with 0.5% saponin (Sigma). Intracellular EhLPPG was stained with mouse-monoclonal antibody EH5 [28] followed by Alexa 594 labeled anti-mouse IgG (Invitrogen). The internalized EhLPPG colocalized with a rat anti- CD107a/LAMP-1 Mab (Southern Biotec) and Alexa 488 labelled anti-rat IgG (Invitrogen). Nuclei were stained with 4‵,6‵-diamidino-2-phenylindole hydrochloride (DAPI). All stainings were visualized with a Leica DRM confocal microscope and OpenLab software (Improvison. Inc).

### Statistics

Statistics were performed using Prism statistical software (GraphPad) unpaired, one-way, non parametric Mann-Whitney U tests, the ANOVA, Dunnett and the student t test.
